# Dr. A. W. Harlan Retires

**Published:** 1902-03

**Authors:** 


### DR. A. W. HARLAN RETIRES.

THE announcement that the editor of the *Dental Review* retires because the service is "not remunerative" will be received with general regret. Dr. Harlan has made the *Review* the leading dental publication in the middle West, and while it has not been prolific in editorial opinions, its influence has always been for an advanced professional standard.

Dr. C. N. Johnson succeeds to the editorial chair. The *Review* under his management will have no uncertain sound. He is recognized throughout the dental profession as a man having ideas and ready at all times to defend them. With this character, and previous editorial experience, he will give renewed life to the journal, and may we be permitted to express the hope that he will rise superior to the trade influences with which he is necessarily surrounded?

